# Clinical characteristics, treatment patterns, and survival in mantle cell lymphoma: a real-world cohort

**DOI:** 10.3389/fonc.2026.1756632

**Published:** 2026-01-30

**Authors:** Cesar de la Hoz, Alexandra Hurtado-Otiz, Maricel Licht-Ardila, Ana Cantillo, Andrea Silva, Leidy Herrera, Paola Alvarez, Olga Daniela Vega Jiménez, Jaiver Fonseca, Alejandra Mendoza-Monsalve, Jhon Alexander Avila, Edgar Fabián Manrique-Hernández

**Affiliations:** 1 Department of Hematology, Hospital Internacional de Colombia HIC- Fundación Cardiovascular de Colombia FCV- Fundación Universitaria FCV, Piedecuesta, Santander, Colombia; 2 Epidemiology Department, Hospital Internacional de Colombia HIC- Fundación Cardiovascular de Colombia FCV- Fundación Universitaria FCV, Piedecuesta, Santander, Colombia

**Keywords:** Colombia, lymphoma, mantle-cell, prognosis, survival analysis, treatment outcome

## Abstract

**Introduction:**

Mantle cell lymphoma is a rare, typically aggressive B-cell non-Hodgkin lymphoma that often presents at an advanced stage and carries substantial long-term mortality despite therapeutic advances. Multiple clinical, biological, and treatment-related factors may influence survival in these patients.

**Objective:**

To describe the clinical characteristics, treatment patterns, and overall survival of patients with mantle cell lymphoma at a high-complexity referral center in a middle-income country.

**Methods:**

This retrospective cohort included adult patients diagnosed and managed at a high-complexity referral center in Colombia between January 2016 and October 2025. Overall survival was the primary outcome. Survival estimates were obtained using Kaplan–Meier curves and compared with log-rank tests.

**Results:**

A total of 41 patients were included, with 43.9% aged <65 years. Baseline clinical, laboratory, immunophenotypic, and cytological characteristics were similar across age groups, with most patients presenting with advanced disease, including Ann Arbor stage IV (66.7% vs. 72.3%) and high-risk MIPI classification (88.9% vs. 100%). First-line treatment differed by age, with younger patients more frequently receiving Nordic regimens and older patients receiving R-bendamustine; however, response rates were comparable. Overall survival did not differ significantly by sex, age group, Ann Arbor stage, or LDH levels. In contrast, MIPI category significantly predicted survival (log-rank p=0.017).

**Conclusion:**

This real-world cohort describes patients with mantle cell lymphoma presenting predominantly with advanced-stage, high-risk disease. Treatment approaches differed by age, with comparable overall responses across groups. The findings suggest that prognostic indices and functional status assessment may aid outcome evaluation in routine practice.

## Introduction

Mantle cell lymphoma (MCL) is an uncommon subtype of B-cell non-Hodgkin lymphoma, accounting for approximately 3%–6% of all cases (1). It is typically diagnosed in older adults, with a median age of 60–70 years, and frequently presents at an advanced stage with widespread nodal and extranodal involvement (2). Historically, the prognosis of MCL has been unfavorable. In a large population-based Finnish cohort, the median overall survival (OS) was 80 months and the 5- and 10-year OS rates were 58% and 32%, respectively, underscoring the substantial long-term mortality associated with this disease (3). More recent real-world series, however, report improved outcomes, particularly among patients treated with intensive immunochemotherapy and novel targeted agents (4).

Several clinical and biological variables, such as age, performance status, serum lactate dehydrogenase (LDH), leukocyte count, and proliferative index, have been incorporated into prognostic tools including the Mantle Cell Lymphoma International Prognostic Index (MIPI) (5–7). Nonetheless, these indices do not fully reflect the biological and clinical heterogeneity of MCL. Additional factors related to comorbidities, treatment intensity and sequencing, disease burden at diagnosis, and institutional practice patterns may also influence patient outcomes (8). Moreover, most available prognostic evidence derives from clinical trials or reference centers in high-income countries, often based on highly selected patient populations, which limits the generalizability of these findings to routine practice and to settings with different resource constraints (9).

In this context, evaluating survival patterns in unselected cohorts treated in high-complexity institutions offers an opportunity to generate evidence that is more representative of real-world practice, particularly in middle-income countries. Understanding the clinical, demographic, and therapeutic characteristics associated with survival may refine risk stratification and inform context-adapted management strategies. Therefore, this study aims to describe the clinical characteristics, treatment patterns, and overall survival of patients with mantle cell lymphoma treated in routine practice at a high-complexity referral center in a middle-income country, providing real-world evidence from a Latin American setting.

## Methods

This retrospective, descriptive cohort study included adult patients (≥18 years) with a diagnosis of mantle cell lymphoma (MCL). Consecutive patients diagnosed and treated at a high-complexity referral center between January 2016 and October 2025 were included. The diagnosis of MCL was established according to the World Health Organization Classification of Tumours of Haematopoietic and Lymphoid Tissues (10), based on histopathological confirmation from an excisional lymph node biopsy.

Baseline data were extracted from Electronic Health Records and included a comprehensive set of sociodemographic, clinical, laboratory, immunophenotypic, cytological, and treatment-related variables. Sociodemographic variables comprised sex, age, and insurance regimen. Clinical variables included the presence of B symptoms (defined as unexplained fever >38°C, drenching night sweats, and unintentional weight loss ≥10% over six months) (11), Ann Arbor stage (12), the simplified Mantle Cell Lymphoma International Prognostic Index (MIPI) (5), Eastern Cooperative Oncology Group (ECOG) (13) performance status, extranodal involvement, and affected organs. The simplified Mantle Cell Lymphoma International Prognostic Index (MIPI) was calculated when all required components were available.

Laboratory variables included hemoglobin concentration, white blood cell count, platelet count, and lactate dehydrogenase (LDH) levels. Cytological morphology was classified as classic, blastic, pleomorphic, or nodular. Immunophenotypic and molecular markers, when available, included CD5, CD23, cyclin D1, SOX11, and TP53. Treatment-related variables included first-, second-, and third-line regimens, number of cycles administered, use of maintenance therapy and agent (R-CHOP, R-bendamustine, BRIGHT, R-hyperCVAD, ibrutinib, R-CVP, R-ESHAP, R-BAC, R-DHAP, rituximab, other regimens). Autologous Bone Marrow Transplantation (ABMT), and best response achieved (complete response, partial response, stable disease, or progression). Data was collected and managed using a secure institutional electronic database.

Patients were descriptively stratified by age using a cutoff of 65 years (<65 vs. ≥65 years) to facilitate comparison of baseline characteristics, treatment patterns, and observed outcomes. This age-based stratification was considered exploratory and descriptive, given the small sample size and the inclusion of age as a component of the MIPI score. The primary outcome was overall survival (OS), defined as the time from diagnosis to death from any cause or last documented follow-up, with surviving patients censored at the latter date. Treatment response rates were reported descriptively as secondary outcomes.

Analyses were stratified using an age cutoff of 65 years, comparing clinical, biological, and outcome variables between patients younger than 65 and those aged 65 years or older, the primary endpoint was long-term overall survival (14). Secondary endpoints included treatment response rates. Associations between baseline variables, including sociodemographic characteristics, clinical presentation, laboratory values, immunophenotypic markers, cytological subtype, and treatment characteristics, and treatment response were explored. OS was defined as the interval from diagnosis date to death from any cause or last documented follow-up, with surviving patients censored at the latter date.

Assessment of treatment response: Treatment response was evaluated according to standard institutional criteria, primarily based on the Lugano and Cheson guidelines (15), using available imaging and laboratory data from the medical records. Due to the retrospective design of the study, some assessments were not performed uniformly for all patients. For transparency, the number of patients with available data for each response category is reported.

### Statistical analysis

Categorical variables were summarized as frequencies and percentages, and continuous variables as medians with interquartile ranges (IQRs). Comparisons between age groups were performed using Fisher’s exact test for categorical variables and the Mann–Whitney U test for continuous variables. For categorical variables that included categories reflecting unavailable or not performed data, p values were calculated exclusively using categories with available information, and categories corresponding to missing or not performed data were excluded from hypothesis testing but retained in descriptive reporting. Overall survival was described using Kaplan–Meier curves, and survival distributions were compared using the log-rank test for descriptive purposes. Analyses were conducted on a complete-case, per-variable basis, and the extent of missing data for each variable is explicitly reported. A two-sided p value <0.05 was considered statistically significant. All analyses were performed using Stata version 16 (StataCorp LLC, College Station, TX).

### Ethical considerations

The protocol received approval from the Institutional Research Ethics Committee (2024-07461-23). Procedures complied with national regulations and the Declaration of Helsinki. Data were anonymized prior to analysis.

## Results

Among the 41 patients with MCL, 18 (43.9%) were younger than 65 years and 23 (56.1%) were aged ≥65 years. Sex distribution was similar between groups (male: 72.2% vs. 78.3%, p=0.724). About insurance regimen (subsidized: 50.0% vs. 56.5%, p=0.758), advanced Ann-Arbor stage IV disease was prevalent in both groups (66.7% vs. 72.3%, p=0.480). Nearly all patients were classified as high-risk by the MIPI score (88.9% vs. 100%, p=0.187). Functional status showed little variation, with ECOG ≥2 in 43.4% of younger patients and 22.2% of older patients (p=0.182) (**Table 1**).

**Table 1:** Baseline clinical, biological, and treatment characteristics of patients with mantle cell lymphoma, stratified by age (<65 vs ≥65 years).

Variable	Category	<65 *n* (%)	≥65 *n* (%)	Total *n* (%)	*p-value*
		18 (43.9)	23 (56.1)	41 (100)	
Sex	Male	13 (72.2)	18 (78.3)	31 (75.6)	0.724
	Female	5 (27.8)	5 (21.7)	10 (24.4)	
Insurance Regimen	Subsidized	9 (50.0)	13 (56.5)	22 (53.7)	0.758
	Contributory	9 (50.0)	10 (43.5)	19 (46.3)	
B Symptoms	No	5 (27.8)	3 (13.0)	8 (19.5)	0.267
	Yes	13 (72.2)	20 (87.0)	33 (80.5)	
Ann Arbor Stage	I	2 (11.1)	1 (4.3)	3 (7.3)	0.480
	II	0 (0)	0 (0)	0 (0)	
	III	0 (0)	2 (8.7)	2 (4.9)	
	IV	12 (66.7)	18 (72.3)	30 (73.2)	
	Not Performed	4 (22.2)	2 (8.7)	6 (14.6)	
MIPI	Low	1 (5.56)	0 (0.00)	1 (2.4)	0.187
	Intermediate	1 (5.56)	0 (0.00)	1 (2.4)	
	High	16 (88.9)	23 (100)	39 (95.1)	
ECOG	0	3 (16.7)	1 (4.3)	4 (9.8)	0.182
	1	9 (50.0)	9 (39.1)	18 (43.9)	
	2	2 (11.1)	5 (21.7)	7 (17.1)	
	3	0 (0)	4 (17.4)	4 (9.8)	
	4	2 (11.1)	1 (4.3)	3 (7.3)	
	No information	4 (22.2)	3 (13.0)	5 (12.2)	
Extranodal Involvement (Yes/No)	No	5 (27.8)	3 (13.0)	8 (19.5)	0.674
	Yes	13 (72.2)	20 (87.0)	33 (80.5)	
Affected organs	None	4 (22.2)	2 (8.7)	6 (14.6)	0.465
	Gastrointestinal tract	0 (0.0)	1 (4.3)	1 (2.4)	
	Central nervous system	1 (5.6)	4 (17.5)	5 (12.2)	
	Bone marrow	0 (0.0)	1 (4.3)	1 (2.4)	
	Spleen	2 (11.1)	0 (0.0)	2 (4.9)	
	Liver	11 (61.1)	15 (65.2)	26 (63.4)	
CD5	Negative	0 (0)	1 (4.3)	1 (2.4)	1
	Positive	15 (83.3)	16 (69.6)	31 (75.6)	
	Not performed	3 (16.7)	6 (26.1)	9 (22.0)	
CD23	Negative	11 (61.1)	11 (47.8)	22 (53.7)	1
	Positive	1 (5.6)	2 (8.7)	3 (7.3)	
	Not performed	3 (16.7)	4 (17.4)	16 (39.0)	
Cyclin D1	Negative	1 (5.6)	3 (13.0)	4 (9.8)	0.603
	Positive	14 (77.8)	14 (60.9)	28 (68.3)	
	Not performed	3 (16.7)	6 (26.1)	9 (22.0)	
ABMT/Transplant	No	4 (22.2)	6 (26.1)	10 (24.4)	0.061
	Yes	5 (27.8)	0 (0.0)	5 (12.2)	
	It has an indication	1 (5.6)	2 (8.7)	3 (7.3)	
	Not information	9 (44.4)	17 (65.2)	26 (56.1)	
Final Status	Alive without disease	10 (55.6)	11 (47.8)	21 (51.2)	0.919
	Alive with disease	5 (27.8)	7 (30.4)	12 (29.3)	
	Deceased	3 (16.7)	5 (21.7)	8 (19.5)	
TP 53	Positive	0 (0.0)	1 (4.3)	1 (2.4)	0.563
	Negative	14 (77.8)	17 (73.9)	31 (75.6)	
	Not performed	4 (22.2)	5 (21.7)	9 (22.0)	
SOX 11	Negative	1 (5.6)	2 (8.7)	3 (7.3)	0.847
	Positive	2 (11.1)	5 (21.7)	7 (17.1)	
	Not performed	15 (83.3)	16 (69.6)	31 (75.6)	
Cytological subtypes	Classic	9 (50.0)	16 (69.6)	25 (61.0)	0.153
	Blastic	2 (11.1)	1 (4.3)	3 (7.3)	
	Pleomorphic	2 (11.1)	0 (0.0)	2 (4.9)	
	Nodular	0 (0.0)	2 (8.7)	2 (4.9)	
	Not information	5 (27.8)	4 (17.4)	9 (22.0)	
Hemoglobin	g/dL	11.4 (9.3-14.6)	10.5 (8.9-12.8)	10.9 (9.2-12.9)	0.212
Leukocytes	x10^3/µL	6.62 (4.6-11.4)	12.52 (7.4-32.5)	8.87 (6.01-13.8)	0.26
Platelets	x10^3/µL	195 (139-248)	169 (113-200)	182 (126-215)	0.193
LDH	Normal	9 (50.0)	9 (39.1)	18 (43.9)	0.484
	High	9 (50.0)	14 (60.9)	23 (56.1)	

Categorical variables are presented as *n (%)*, and continuous variables (hemoglobin, leukocyte count, and platelet count) are presented as median (interquartile range, IQR). Percentages are calculated within each age group. For variables with unavailable, not performed, or not reported data, these categories are shown descriptively; however, p values were calculated exclusively using categories with available information, excluding missing or not performed categories from hypothesis testing. Comparisons between age groups were performed using Fisher’s exact test for categorical variables and the Mann–Whitney U test for continuous variables. A two-sided *p* value <0.05 was considered statistically significant. ECOG, Eastern Cooperative Oncology Group performance status; MIPI, Mantle Cell Lymphoma International Prognostic Index; ABMT, Autologous Bone Marrow Transplantation; CD, Cluster of Differentiation; LDH, lactate dehydrogenase; IQR, interquartile range.

Extranodal involvement was common across both strata (72.2% vs. 87.0%, p=0.674), predominantly affecting the liver (63.4% overall, p=0.465). Immunophenotypic markers were comparable, including CD5 positivity (83.3% vs. 69.6%) and cyclin D1 expression (77.8% vs. 60.9%, p=0.603). Laboratory parameters were similar, including median hemoglobin (11.4 vs. 10.5 g/dL, p=0.212), platelet count (195 vs. 169×10³/µL, p=0.193), and LDH elevation (50.0% vs. 60.9%, p=0.484) (**Table 1**).

Regarding cytological morphology, the classic subtype predominated across both age categories, representing 61.0% of the overall cohort (50.0% among younger patients and 69.6% among older patients, p = 0.153). Blastic morphology accounted for 7.3% of cases, with a slightly higher frequency in the younger group (11.1% vs. 4.3%). The pleomorphic variant was observed exclusively in younger patients (11.1%), whereas the nodular subtype appeared only in the older category (8.7%) (**Table 1**).

**Figure 1:**
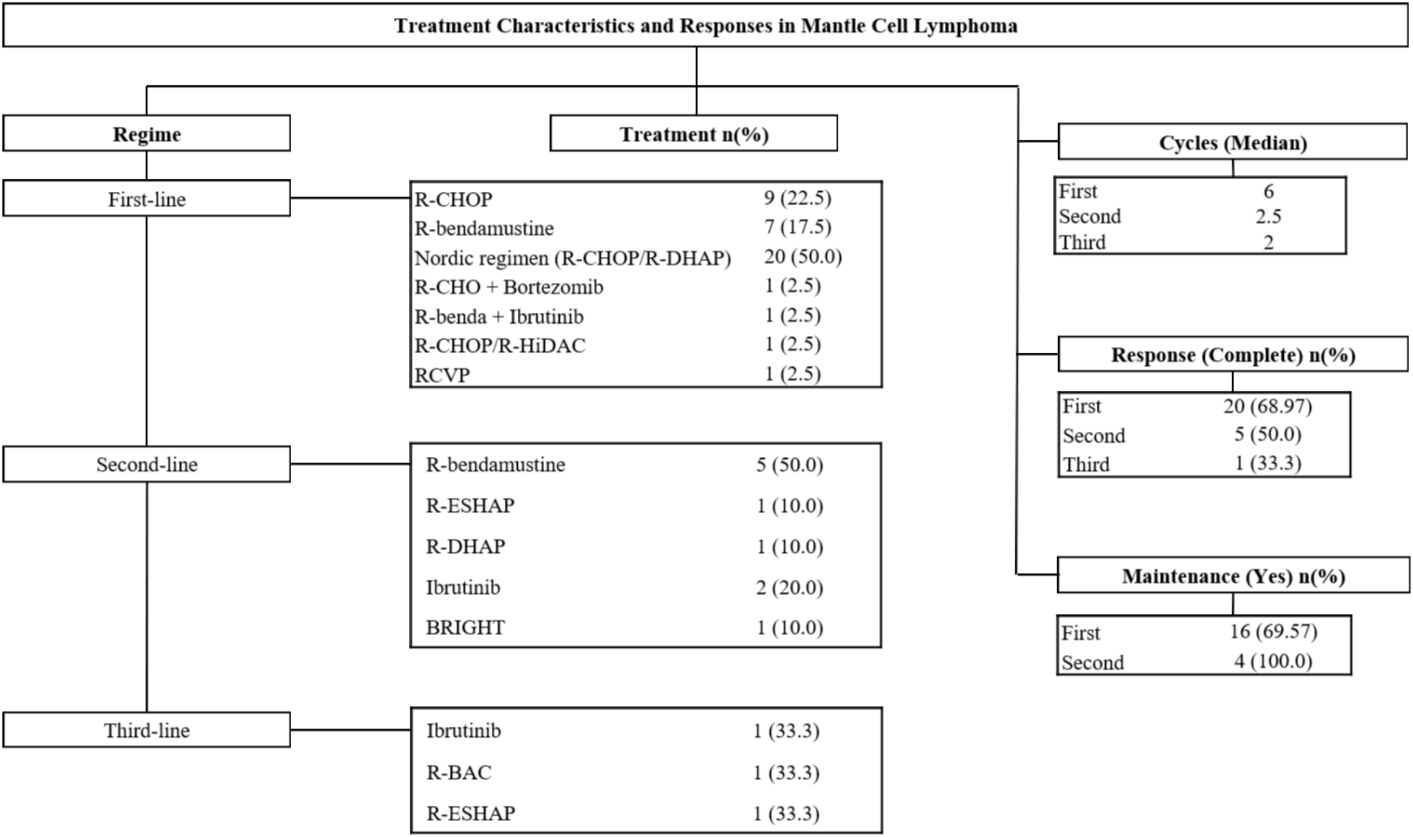
Treatment characteristics and responses in mantle cell lymphoma stratified by line.

When comparing first-line treatment between patients younger than 65 years and those aged 65 years or older. Younger patients more frequently received Nordic regimen (R-CHOP/R-DHAP) (76.5% vs. 30.4%), whereas older patients were more commonly treated with R-bendamustine (30.4%) and other less intensive alternatives. The first-line treatment response was similar across age groups, with comparable complete response rates (75.0% in <65 vs. 64.7% in ≥65). Likewise, no differences were identified in the use of maintenance therapy (62.5% in <65 vs. 73.3% in ≥65; p = 0.657), with rituximab being the predominant maintenance agent in both cohorts (86.7%) (**Figure 1**).

In second-line treatment patterns between patients younger than 65 years and those aged 65 years or older (p=0.014). Older patients received R-bendamustine more frequently (83.3%; n=5), whereas younger patients were treated with ibrutinib (50.0%; n=2) or other alternative regimens. Complete responses occurred in 25.0% (n=1) of younger patients and 66.7% (n=4) of older patients. In third-line therapy, only three patients were treated (<65: n=2; ≥65: n=1). Among younger patients, half received R-BAC and half received R-ESHAP, whereas the only older patient received ibrutinib. Treatment responses were evenly split: one younger patient achieved complete response (50%), while the older patient experienced persistent disease (**Figure 1**).

**Figure 2:**
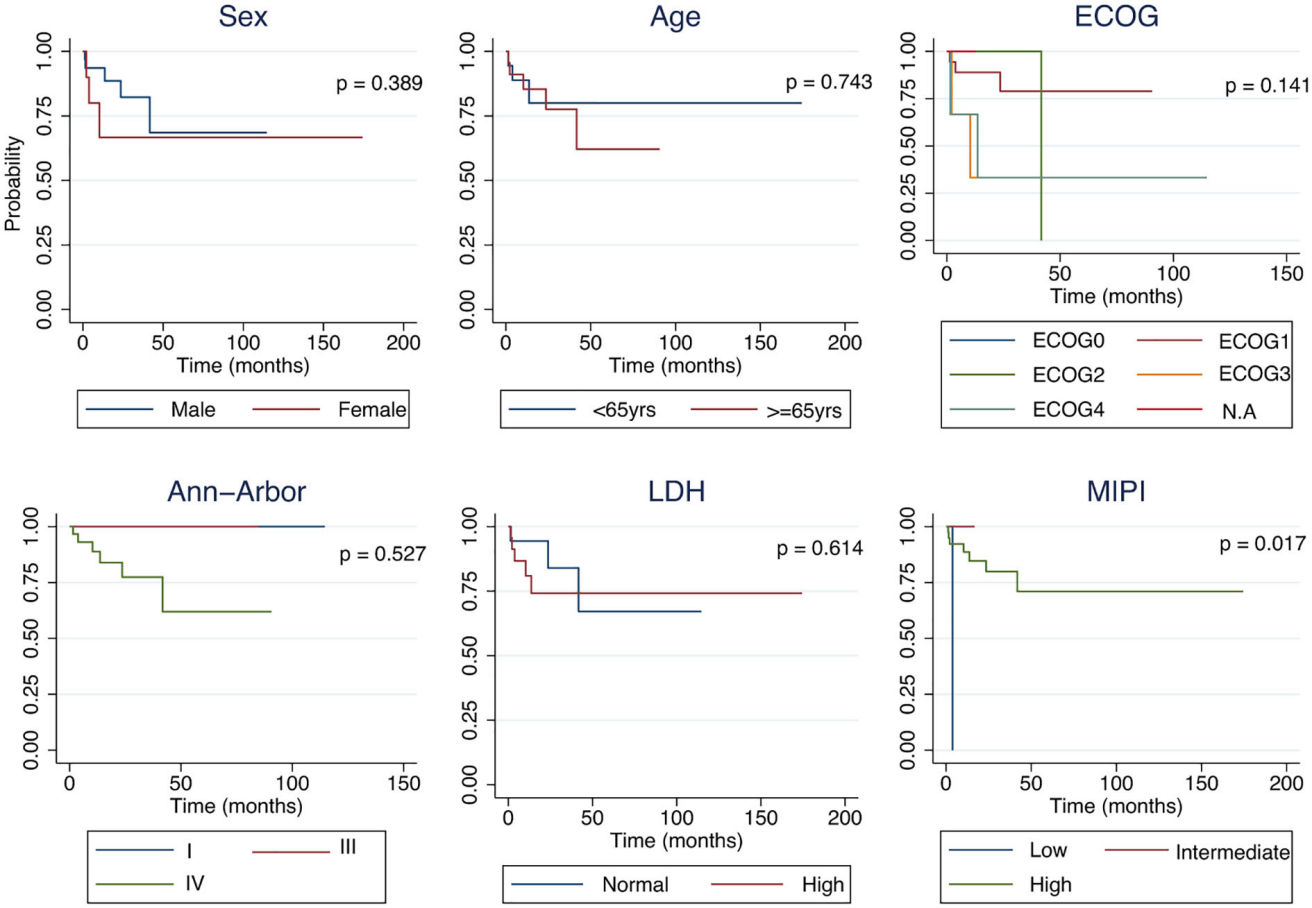
Kaplan–Meier curves showing overall survival stratified by sex, age group, ECOG performance status, Ann Arbor stage, LDH levels, and MIPI risk category.

Overall survival (OS) by sex, among men, survival probabilities were 93.6% at 1 year, 82.3% at 3 years, and 68.6% at 5 years, whereas women had a 1-year survival of 66.7%, which remained unchanged at 3 and 5 years. Survival by age group was also similar, patients <65 years had survival rates of 88.9% at 1 and 3 years, 80.0% at 5 and 10 years, while those ≥65 years showed 85.4% at 1 year, 77.6% at 3 years, and 62.1% at 5 years, remaining stable at 62.1% at the latest follow-up. A gradient was observed across ECOG categories, patients with ECOG 0 and 2 maintained 100% survival throughout most of follow-up, whereas ECOG 1 decreased from 88.9% at 1–3 years to 79.0% later. ECOG 3–4 were related with poorer outcomes, with survival falling to 66.7% early and 33.3% within the first year. Survival did not differ by Ann Arbor stage (log-rank p = 0.527) or LDH level (log-rank p = 0.614). In contrast, MIPI (log-rank p = 0.017): no deaths occurred in the intermediate-risk groups, whereas high-risk patients declined from 97% early to 71% at later follow-up (**Figure 2**).

## Discussion

This cohort study provides a comprehensive characterization of clinical features, treatment patterns, and survival outcomes among patients with MCL treated at a high-complexity referral center in a middle-income country. The study captures routine clinical practice over nearly a decade, offering valuable insights into prognostic determinants outside the controlled environment of clinical trials, an area where evidence remains limited, particularly in Latin America.

Our population exhibited a predominantly high-risk profile, with 95% of patients classified as high-risk by MIPI and 85.7% presenting with stage IV disease, figures higher than those reported in many European and North American series (5, 16). The uniformly high-risk profile observed in our cohort may reflect health system and referral patterns. As a tertiary referral center, we receive a higher proportion of complex and advanced presentations. In addition, delays in access to specialized care and diagnostic evaluation in routine clinical practice may contribute to late-stage diagnosis and higher observed tumor burden. These contextual factors may partially explain the apparent clinical homogeneity of risk in this dataset when compared with broader population-based cohorts (16, 17).

The long inclusion period of our cohort (2016–2025) encompasses significant changes in the management of mantle cell lymphoma, including increased access to BTK inhibitors and evolving autologous transplant strategies. All consecutive adult patients were included, even those with incomplete records, which introduces some temporal heterogeneity that may influence comparisons of outcomes over time. Acknowledging this context allows the findings to be interpreted as representative of real-world clinical practice in a high-complexity center in a middle-income country and underscores the need for multicenter studies that account for evolving treatment strategies.

Baseline characteristics, including ECOG performance status, LDH levels, and immunophenotypic markers, did not differ significantly between age groups, suggesting a clinically homogeneous and uniformly advanced disease at diagnosis (18). This pattern is consistent with regional reports in which delayed diagnosis and limited access to early evaluation contribute to late-stage presentation (17).

Beyond clinical presentation, mantle cell lymphoma is characterized by biological heterogeneity that influences prognosis. High-risk cytological variants, such as blastoid and pleomorphic morphology, have been associated with inferior outcomes and treatment resistance (19). In our cohort, these variants were infrequent, limiting their contribution to survival differences. Similarly, TP53 alterations and SOX11 expression define biologically distinct subgroups with important prognostic implications in MCL (20, 21). Although molecular data were incomplete in our series, the established relevance of these biomarkers highlights the need for more systematic biological characterization in real-world practice.

Sex showed a non-significant trend toward better OS in males, in line with population-based studies where sex rarely emerges as an independent prognostic factor once age, performance status, and treatment are accounted for. Similarly, we did not observe a statistically significant difference in OS between patients <65 and ≥65 years (22). Although older age has been associated with poorer outcomes in large multicenter cohorts, differences in our study may have been attenuated by sample size, selection of fitter older patients, and the use of adapted but effective treatment strategies (23). As expected, younger patients more often received intensive Nordic regimens, whereas older patients were treated with R-bendamustine; nonetheless, first-line response rates were comparable, echoing evidence that less intensive regimens can provide adequate disease control in carefully selected older or frail patients (24). This observation should be interpreted with caution and considered descriptive, particularly given the overlap between chronological age and components of the MIPI score. This observation should be interpreted with caution and considered descriptive, particularly given the overlap between chronological age and components of the MIPI score.

ECOG performance status showed a clear clinical gradient: patients with ECOG 0–2 maintained favorable long-term survival, whereas those with ECOG 3–4 experienced markedly higher early mortality (3, 25). Although this association did not reach statistical significance, likely due to limited power, the pattern reinforces the central role of functional status as a prognostic marker in MCL and supports its continued use in risk assessment (26). In contrast, Ann Arbor stage and LDH did not significantly stratify OS, which is not unexpected given that most patients had stage IV disease and that the prognostic value of these traditional variables often diminishes once more integrative indices, such as MIPI (27).

In this context, MIPI emerged as the most informative prognostic indicator in our cohort. Consistent with the findings of Hoster et al., patients classified as low- or intermediate-risk showed better survival, while those in the high-risk category experienced a progressive decline in survival (5). However, since nearly all patients in our cohort were classified as high-risk, the discriminatory capacity of MIPI within this population is limited compared to multicenter European and North American series (28). Therefore, conclusions regarding independent associations of other clinical or laboratory variables with survival are not possible due to insufficient statistical power (28, 29). Absence of statistical significance should not be interpreted as evidence of no association (29). Consequently, our discussion emphasizes observed trends and patterns rather than definitive negative findings, providing a realistic perspective on the utility of MIPI in predominantly high-risk settings.

In our cohort, therapeutic decisions were guided by patients’ functional status and transplant eligibility. Intensive regimens, such as the Nordic protocols, were preferentially offered to younger or fit patients, whereas older or frail patients received adapted schemes, such as R-bendamustine. Maintenance therapy, primarily with rituximab, was administered at the physician’s discretion and according to patient tolerance. Access to novel agents, including ibrutinib, was influenced by availability and institutional protocols. These considerations reflect real-world practice patterns and provide context for the observed treatment heterogeneity, highlighting the challenges and decision-making processes in a middle-income setting.

Clinically, these results support the cautious application of MIPI for risk stratification, treatment planning, and follow-up intensity, while recognizing its limitations in predominantly high-risk populations. They also suggest that chronological age alone should not preclude consideration of intensive therapy in fit patients, and that careful evaluation and optimization of performance status may be critical to improving outcomes. From a translational perspective, future studies integrating biological insights from cell line–derived models and tumor microenvironment analyses may help link clinical outcomes with mechanisms of cell migration, drug resistance, focal adhesion kinase signaling, NF-kappa B activation, and gene expression patterns relevant to mantle cell lymphoma in both female and male patients (30). From a research perspective, these findings underscore the need for larger, multicenter studies to explore additional prognostic markers and to validate patterns observed in real-world Latin American settings.

### Limitations

The modest sample size limits statistical power, particularly for subgroup comparisons. The retrospective design may introduce information bias related to heterogeneous clinical records; however, the extent of missing data for relevant variables was explicitly reported. As a single-center study, generalizability may be limited to settings with similar referral patterns and treatment availability. The long inclusion period (2016–2025) spans changes in treatment strategies, introducing temporal heterogeneity. Potential observational biases, including immortal time bias, lead-time bias, and survivorship bias, cannot be excluded. In addition, patients diagnosed closer to 2025 had shorter follow-up, which may underestimate late events. Therefore, the findings should be interpreted as descriptive and hypothesis-generating.

## Conclusion

This real-world study describes the clinical characteristics, treatment patterns, and survival outcomes of patients with mantle cell lymphoma treated at a high-complexity referral center in Colombia. Most patients presented with advanced-stage disease and high-risk MIPI profiles, reflecting a substantial tumor burden at diagnosis. Therapeutic approaches varied by age, with more intensive regimens used in younger patients and adapted treatments in older individuals, yielding overall comparable responses. Taken together, these findings suggest that standardized prognostic indices and functional status assessment may be useful tools for describing outcomes and supporting clinical decision-making in real-world settings. This approach is particularly relevant in middle-income countries, where access to advanced molecular characterization is limited, and underscores the need for larger multicenter studies to confirm and extend these observations.

## Data Availability

The original contributions presented in the study are included in the article/supplementary material. Further inquiries can be directed to the corresponding author.
